# Methyl *N*′-[(*E*)-1-phenyl­ethyl­idene]hydrazinecarboxyl­ate

**DOI:** 10.1107/S1600536808019259

**Published:** 2008-07-05

**Authors:** Xiang-Wei Cheng

**Affiliations:** a Zhejiang Police College Experience Center, Zhejiang Police College, Hangzhou 310053, People’s Republic of China

## Abstract

The mol­ecule of the title compound, C_10_H_12_N_2_O_2_, adopts a *trans* configuration with respect to the C=N bond. The dihedral angle between the phenyl ring and the hydrazine carboxylic acid mean plane is 25.23 (9)°. In the crystal structure, mol­ecules are linked into chains by N—H⋯O hydrogen bonds and C—H⋯π inter­actions.

## Related literature

For a related structure and background, see: Cheng (2008 ▶).
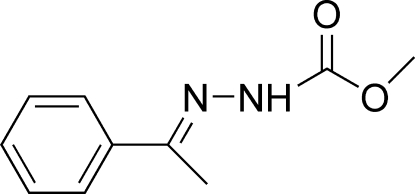


## Experimental

### 

#### Crystal data

C_10_H_12_N_2_O_2_
*M* *_r_* = 192.22
Orthorhombic, 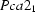

*a* = 6.6733 (5) Å
*b* = 19.8940 (14) Å
*c* = 7.7254 (5) Å
*V* = 1025.61 (12) Å^3^
*Z* = 4
Mo *K*α radiation
μ = 0.09 mm^−1^
*T* = 123 (2) K
0.26 × 0.25 × 0.23 mm

#### Data collection

Bruker SMART CCD diffractometer
Absorption correction: multi-scan (*SADABS*; Bruker, 2002 ▶) *T*_min_ = 0.965, *T*_max_ = 0.968
10169 measured reflections
971 independent reflections
935 reflections with *I* > 2σ(*I*)
*R*_int_ = 0.022

#### Refinement

*R*[*F* ^2^ > 2σ(*F* ^2^)] = 0.028
*wR*(*F* ^2^) = 0.076
*S* = 1.14
971 reflections
143 parameters
1 restraint
H atoms treated by a mixture of independent and constrained refinement
Δρ_max_ = 0.11 e Å^−3^
Δρ_min_ = −0.09 e Å^−3^

### 

Data collection: *SMART* (Bruker, 2002 ▶); cell refinement: *SAINT* (Bruker, 2002 ▶); data reduction: *SAINT*; program(s) used to solve structure: *SHELXS97* (Sheldrick, 2008 ▶); program(s) used to refine structure: *SHELXL97* (Sheldrick, 2008 ▶); molecular graphics: *SHELXTL* (Sheldrick, 2008 ▶); software used to prepare material for publication: *SHELXTL*.

## Supplementary Material

Crystal structure: contains datablocks I, global. DOI: 10.1107/S1600536808019259/hb2751sup1.cif

Structure factors: contains datablocks I. DOI: 10.1107/S1600536808019259/hb2751Isup2.hkl

Additional supplementary materials:  crystallographic information; 3D view; checkCIF report

## Figures and Tables

**Table 1:** Hydrogen-bond geometry (Å, °) *Cg*1 is the centroid of the C1–C6 ring.

*D*—H⋯*A*	*D*—H	H⋯*A*	*D*⋯*A*	*D*—H⋯*A*
N2—H10⋯O1^i^	0.84 (4)	2.16 (4)	2.977 (2)	167
C2—H2*A*⋯*Cg*1^ii^	0.95	2.96	3.827 (2)	156
C5—H5⋯*Cg*1^iii^	0.95	2.88	3.753 (2)	156

Symmetry codes: (i) 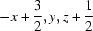; (ii) 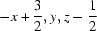; (iii) 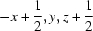.

**Fig. 1.**
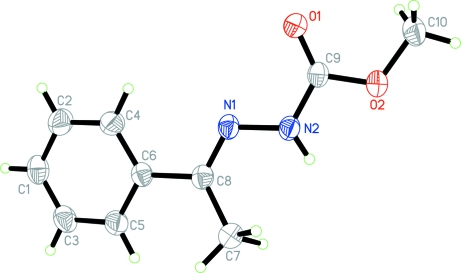
The molecular structure of (I), showing 30% probability displacement ellipsoids for the non-hydrogen atoms.

**Fig. 2.**
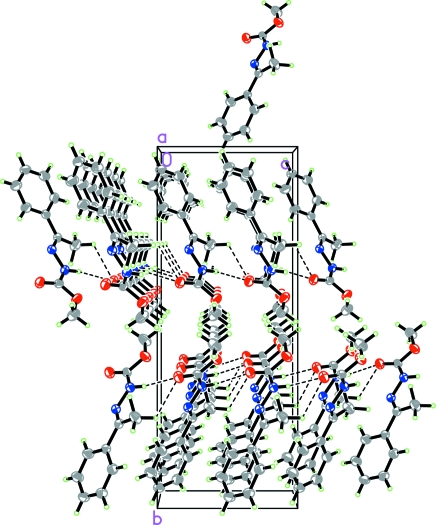
Crystal packing of (I), viewed approximately down the a axis. Dashed lines indicate hydrogen bonds.

## supplementary crystallographic information

### Comment

As part of our ongoing studies of benzaldehydehydrazone derivatives (Cheng, 2008), we now report the synthesis and structure of the title compound, (I).

The title molecule (Fig. 1) adopts a *trans* configuration with respect to the C=N bond. The C9/C10/N2/O1/O2 plane of the hydrazine carboxylic acid methyl ester group is slightly twisted away from the attached ring. The dihedral angle between the C1—C6 ring and the C9/C10/N2/O1/O2 plane is 25.23 (9)°. Otherwise, the bond lengths and angles ij (I) agree with those observed for (*E*)-methyl *N*'-(4-hydroxybenzylidene) hydrazinecarboxylate (Cheng, 2008).

In the crystal structure, N–H···O hydrogen bonds and C–H···π interactions (Table 1) link the molecules into chains (Fig. 2).

### Experimental

Acetophenone (1.2 g, 0.01 mol) and methyl hydrazinecarboxylate (0.9 g, 0.01 mol) were dissolved in stirred methanol (20 ml) and left for 2 h at room temperature. The resulting solid was filtered off and recrystallized from ethanol to give the title compound in 90% yield. Colourless blocks of (I) were obtained by slow evaporation of a ethanol solution at room temperature (m.p. 450–452 K).

### Refinement

Anomalous dispersion was negligible and Friedel pairs were merged before refinement. The H atoms attached to C7 and N2 were located in a difference map and their positions and U_iso_ values were freely refined. The other H atoms were geometrically placed (C—H = 0.95-0.98Å) and refined as riding with *U*_iso_(H) = 1.2–1.5*U*_eq_(C).

### Crystal data

**Table:**

C_10_H_12_N_2_O_2_	*F*_000_ = 408
*M**_r_* = 192.22	*D*_x_ = 1.245 Mg m^−^^3^
Orthorhombic, *P**c**a*2_1_	Mo *K*α radiation λ = 0.71073 Å
Hall symbol: P 2c -2ac	Cell parameters from 971 reflections
*a* = 6.6733 (5) Å	θ = 2.0–25.0º
*b* = 19.8940 (14) Å	µ = 0.09 mm^−^^1^
*c* = 7.7254 (5) Å	*T* = 123 (2) K
*V* = 1025.61 (12) Å^3^	Block, colourless
*Z* = 4	0.26 × 0.25 × 0.23 mm

### Data collection

**Table:**

Bruker SMART CCD diffractometer	971 independent reflections
Radiation source: fine-focus sealed tube	935 reflections with *I* > 2σ(*I*)
Monochromator: graphite	*R*_int_ = 0.022
*T* = 123(2) K	θ_max_ = 25.0º
ω scans	θ_min_ = 2.1º
Absorption correction: multi-scan(SADABS; Bruker, 2002)	*h* = −7→7
*T*_min_ = 0.965, *T*_max_ = 0.968	*k* = −23→21
10169 measured reflections	*l* = −8→9

### Refinement

**Table:**

Refinement on *F*^2^	Secondary atom site location: difference Fourier map
Least-squares matrix: full	Hydrogen site location: difmap and geom
*R*[*F*^2^ > 2σ(*F*^2^)] = 0.028	H atoms treated by a mixture of independent and constrained refinement
*wR*(*F*^2^) = 0.076	*w* = 1/[σ^2^(*F*_o_^2^) + (0.0468*P*)^2^ + 0.0621*P*] where *P* = (*F*_o_^2^ + 2*F*_c_^2^)/3
*S* = 1.14	(Δ/σ)_max_ = 0.048
971 reflections	Δρ_max_ = 0.11 e Å^−^^3^
143 parameters	Δρ_min_ = −0.08 e Å^−^^3^
1 restraint	Extinction correction: none
Primary atom site location: structure-invariant direct methods	

### Special details

**Table:**

Geometry. All e.s.d.'s (except the e.s.d. in the dihedral angle between two l.s. planes) are estimated using the full covariance matrix. The cell e.s.d.'s are taken into account individually in the estimation of e.s.d.'s in distances, angles and torsion angles; correlations between e.s.d.'s in cell parameters are only used when they are defined by crystal symmetry. An approximate (isotropic) treatment of cell e.s.d.'s is used for estimating e.s.d.'s involving l.s. planes.
Refinement. Refinement of *F*^2^ against ALL reflections. The weighted *R*-factor *wR* and goodness of fit *S* are based on *F*^2^, conventional *R*-factors *R* are based on *F*, with *F* set to zero for negative *F*^2^. The threshold expression of *F*^2^ > σ(*F*^2^) is used only for calculating *R*-factors(gt) *etc*. and is not relevant to the choice of reflections for refinement. *R*-factors based on *F*^2^ are statistically about twice as large as those based on *F*, and *R*- factors based on ALL data will be even larger.

### Fractional atomic coordinates and isotropic or equivalent isotropic displacement parameters (Å^2^)

**Table:**

	*x*	*y*	*z*	*U*_iso_*/*U*_eq_	
O2	0.8998 (2)	0.42251 (7)	0.4199 (2)	0.0606 (4)	
O1	0.9765 (2)	0.37588 (7)	0.1622 (2)	0.0563 (4)	
N2	0.7026 (3)	0.34290 (8)	0.3197 (2)	0.0491 (4)	
N1	0.6763 (2)	0.28503 (7)	0.2224 (2)	0.0472 (4)	
C6	0.5017 (3)	0.18464 (9)	0.1633 (3)	0.0467 (4)	
C8	0.5153 (3)	0.25135 (9)	0.2518 (2)	0.0471 (4)	
C9	0.8707 (3)	0.37937 (9)	0.2891 (3)	0.0448 (4)	
C4	0.6331 (3)	0.16677 (11)	0.0313 (4)	0.0632 (6)	
H4	0.7287	0.1986	−0.0087	0.076*	
C5	0.3635 (3)	0.13717 (10)	0.2157 (3)	0.0610 (5)	
H5	0.2700	0.1480	0.3041	0.073*	
C7	0.3524 (4)	0.27326 (16)	0.3717 (4)	0.0703 (7)	
C2	0.6270 (4)	0.10375 (11)	−0.0424 (4)	0.0688 (7)	
H2A	0.7188	0.0926	−0.1320	0.083*	
C1	0.4902 (4)	0.05692 (10)	0.0119 (4)	0.0634 (6)	
H1	0.4865	0.0135	−0.0393	0.076*	
C10	1.0751 (3)	0.46413 (12)	0.4084 (4)	0.0729 (7)	
H10A	1.0816	0.4937	0.5096	0.109*	
H10B	1.0684	0.4914	0.3029	0.109*	
H10C	1.1949	0.4357	0.4048	0.109*	
C3	0.3594 (4)	0.07359 (11)	0.1404 (4)	0.0665 (6)	
H3	0.2642	0.0414	0.1792	0.080*	
H10	0.642 (4)	0.3456 (12)	0.414 (5)	0.074 (8)*	
H11	0.239 (7)	0.2525 (14)	0.336 (5)	0.100 (9)*	
H12	0.347 (4)	0.3212 (16)	0.373 (5)	0.092 (9)*	
H13	0.400 (8)	0.261 (2)	0.480 (9)	0.16 (2)*	

### Atomic displacement parameters (Å^2^)

**Table:**

	*U*^11^	*U*^22^	*U*^33^	*U*^12^	*U*^13^	*U*^23^
O2	0.0663 (8)	0.0588 (8)	0.0566 (9)	−0.0113 (7)	0.0026 (8)	−0.0173 (7)
O1	0.0578 (7)	0.0621 (9)	0.0488 (8)	−0.0081 (6)	0.0045 (7)	−0.0092 (7)
N2	0.0621 (10)	0.0459 (8)	0.0394 (9)	−0.0059 (7)	0.0030 (8)	−0.0046 (7)
N1	0.0612 (8)	0.0410 (7)	0.0393 (8)	−0.0045 (6)	−0.0020 (7)	−0.0006 (7)
C6	0.0555 (10)	0.0438 (9)	0.0408 (10)	−0.0039 (8)	−0.0054 (8)	0.0032 (8)
C8	0.0562 (10)	0.0469 (9)	0.0381 (10)	−0.0007 (8)	−0.0028 (8)	0.0023 (8)
C9	0.0507 (9)	0.0410 (9)	0.0428 (10)	0.0029 (7)	−0.0068 (8)	−0.0024 (8)
C4	0.0729 (13)	0.0512 (11)	0.0656 (14)	−0.0112 (10)	0.0152 (12)	−0.0035 (11)
C5	0.0684 (12)	0.0596 (11)	0.0549 (12)	−0.0151 (10)	0.0044 (10)	−0.0012 (11)
C7	0.0636 (13)	0.0744 (17)	0.0728 (18)	−0.0115 (12)	0.0119 (12)	−0.0210 (14)
C2	0.0804 (14)	0.0563 (12)	0.0698 (16)	−0.0010 (11)	0.0110 (13)	−0.0104 (12)
C1	0.0815 (14)	0.0429 (11)	0.0660 (14)	−0.0022 (10)	−0.0125 (12)	−0.0032 (10)
C10	0.0673 (13)	0.0730 (15)	0.0786 (16)	−0.0169 (11)	−0.0033 (13)	−0.0243 (14)
C3	0.0798 (13)	0.0547 (12)	0.0650 (15)	−0.0209 (10)	−0.0032 (12)	0.0034 (11)

### Geometric parameters (Å, °)

**Table:**

O2—C9	1.340 (2)	C5—C3	1.392 (3)
O2—C10	1.436 (2)	C5—H5	0.9500
O1—C9	1.211 (3)	C7—H11	0.91 (4)
N2—C9	1.357 (2)	C7—H12	0.95 (3)
N2—N1	1.386 (2)	C7—H13	0.93 (6)
N2—H10	0.84 (3)	C2—C1	1.370 (3)
N1—C8	1.286 (2)	C2—H2A	0.9500
C6—C5	1.381 (3)	C1—C3	1.363 (4)
C6—C4	1.391 (3)	C1—H1	0.9500
C6—C8	1.496 (3)	C10—H10A	0.9800
C8—C7	1.494 (3)	C10—H10B	0.9800
C4—C2	1.378 (3)	C10—H10C	0.9800
C4—H4	0.9500	C3—H3	0.9500
			
C9—O2—C10	116.16 (17)	C8—C7—H12	109 (2)
C9—N2—N1	117.04 (17)	H11—C7—H12	115 (3)
C9—N2—H10	121.1 (18)	C8—C7—H13	103 (3)
N1—N2—H10	117.8 (18)	H11—C7—H13	116 (4)
C8—N1—N2	116.30 (16)	H12—C7—H13	106 (4)
C5—C6—C4	117.5 (2)	C1—C2—C4	120.8 (2)
C5—C6—C8	120.90 (19)	C1—C2—H2A	119.6
C4—C6—C8	121.57 (17)	C4—C2—H2A	119.6
N1—C8—C7	124.42 (19)	C3—C1—C2	119.0 (2)
N1—C8—C6	115.63 (16)	C3—C1—H1	120.5
C7—C8—C6	119.88 (18)	C2—C1—H1	120.5
O1—C9—O2	124.28 (17)	O2—C10—H10A	109.5
O1—C9—N2	126.33 (18)	O2—C10—H10B	109.5
O2—C9—N2	109.35 (17)	H10A—C10—H10B	109.5
C2—C4—C6	121.1 (2)	O2—C10—H10C	109.5
C2—C4—H4	119.4	H10A—C10—H10C	109.5
C6—C4—H4	119.4	H10B—C10—H10C	109.5
C6—C5—C3	120.8 (2)	C1—C3—C5	120.8 (2)
C6—C5—H5	119.6	C1—C3—H3	119.6
C3—C5—H5	119.6	C5—C3—H3	119.6
C8—C7—H11	107 (2)		
			
C9—N2—N1—C8	−179.38 (17)	N1—N2—C9—O2	−164.20 (15)
N2—N1—C8—C7	4.7 (3)	C5—C6—C4—C2	−0.9 (3)
N2—N1—C8—C6	−172.29 (15)	C8—C6—C4—C2	176.1 (2)
C5—C6—C8—N1	164.0 (2)	C4—C6—C5—C3	1.1 (3)
C4—C6—C8—N1	−12.9 (3)	C8—C6—C5—C3	−175.9 (2)
C5—C6—C8—C7	−13.1 (3)	C6—C4—C2—C1	0.3 (4)
C4—C6—C8—C7	170.0 (3)	C4—C2—C1—C3	0.0 (4)
C10—O2—C9—O1	−3.3 (3)	C2—C1—C3—C5	0.2 (4)
C10—O2—C9—N2	178.85 (18)	C6—C5—C3—C1	−0.8 (4)
N1—N2—C9—O1	18.0 (3)		

### Hydrogen-bond geometry (Å, °)

**Table:**

*D*—H···*A*	*D*—H	H···*A*	*D*···*A*	*D*—H···*A*
N2—H10···O1^i^	0.84 (4)	2.16 (4)	2.977 (2)	167
C2—H2A···Cg1^ii^	0.95	2.96	3.827 (2)	156
C5—H5···Cg1^iii^	0.95	2.88	3.753 (2)	156

Symmetry codes: (i) −*x*+3/2, *y*, *z*+1/2; (ii) −*x*+3/2, *y*, *z*−1/2; (iii) −*x*+1/2, *y*, *z*+1/2.
